# Rapid Sequence Initiation of Device Therapy in Heart Failure

**DOI:** 10.1016/j.jacadv.2023.100316

**Published:** 2023-05-10

**Authors:** Husam M. Salah, Javed Butler, Marat Fudim

**Affiliations:** a Department of Internal Medicine, University of Arkansas for Medical Sciences, Little Rock, Arkansas, USA; b Baylor Scott and White Research Institute, Dallas, Texas, USA; c Department of Medicine, University of Mississippi School of Medicine, Jackson, Mississippi, USA; d Division of Cardiology, Duke University Medical Center, Durham, North Carolina, USA; e Duke Clinical Research Institute, Duke University School of Medicine, Durham, North Carolina, USA

**Keywords:** devices, GDMT, heart failure, HF therapies, sequencing

Despite major advances in drug therapy for heart failure (HF) and efforts to optimize their use amidst a rising trend of HF hospitalizations and excess HF-related morbidity and mortality, most eligible patients do not receive guideline-directed medical therapy (GDMT).^1^ Several device-based therapies are also approved for different phenotypes of HF and are becoming an integral part of HF management, including cardiac resynchronization therapy, implantable cardioverter-defibrillator, transcatheter edge-to-edge mitral repair, cardiac contractility modulation, and baroreflex activation therapy. The conventional approach to HF treatment is to initiate and optimize drugs before considering devices. Indeed, this was a requirement in clinical trials assessing devices in HF, and this approach is based on the temporal development of devices that proceeded the earlier testing and approval of drugs for HF. An alternate approach of initiating device-based therapies prior to or simultaneously with drug therapies in selected patients has not been explored.

Devices offer several advantages over drugs. First, they operate largely independent of patient’s adherence, eliminating nonadherence as a major factor contributing to suboptimal therapy in the real-world practice. Second, they target structural or biological pathways that are not typically amenable to drug therapy. Third, they generally improve hemodynamics with no blood pressure or heart rate lowering effect and have minimal interaction with kidney function; therefore, their use is not interrupted by fluctuations in cardiovascular or kidney status. Fourth, the improvement in hemodynamics associated with their use could enable optimization of drug therapy. Fifth, device-based therapies have no interaction with medications, which is important in patients on polypharmacy.

Several drawbacks for device-based therapies also exist. They require an invasive procedure for implantation with a potential for procedural and device-related complications. They may require maintenance, troubleshooting and replacements if dependent on a battery. The initial cost of devices and their implantation is more than that of drugs. These drawbacks combined with the fact that drugs have been studied in larger populations, are easier to administer, and do not require specialized setting for initiation and management, have conventionally made drugs the first-line approach in patients with HF.

To achieve the full benefits of GDMT, they must first be prescribed by the clinicians, the patients should not have any absolute or relative contraindications, achieve tolerability to target doses, and finally adhere to them in the long run. There are multiple factors that adversely impact the use of long-term consistent drug therapy, including tolerability (eg, kidney function, blood pressure, heart rate, electrolyte imbalance, side effects) and adherence (eg, financial aspects, polypharmacy, lifestyle, and social considerations). Given these, it is no surprise that achieving GDMT in HF is challenging and may in part explain the overall suboptimal use of these drugs and even worse trends in achieve target doses of drugs among eligible patients. For example, in a cohort of ∼15,000 patients with HF with reduced ejection fraction, 70% had a record of medical therapies (a beta-blocker and either angiotensin-converting enzyme inhibitor, angiotensin receptor blocker, or angiotensin receptor-neprilysin inhibitor), and only 57% had concurrent medication fills,^2^ suggesting that 43% of patients with HF with reduced ejection fraction may not be on medical therapies, let alone optimal doses of these therapies. The conventional approach can overlook or delay patients who are not on GDMT (eg, due to nontolerance and/or nonadherence) from being evaluated for device-based therapies. It is important to note that the intolerant patients tend to be sicker with higher risk than the general HF population. Timely therapy, drugs and devices, has the potential to provide clinical and health status benefits in these patients and slow the disease progression. Conventional approaches can enormously delay appropriate device therapy to eligible patients as evaluation for these typically follow GDMT optimization. This is important as patients with HF have up to a 60% loss in life expectancy compared with general population.^3^

A personalized approach to sequencing HF therapies, drugs or devices, may accelerate the delivery of the full potential benefits from them in HF. For example, in patients in whom tolerance is a barrier, an approach that consists of initiating device-based therapies prior to full optimization of GDMT may improve hemodynamics and subsequently enable tolerance to medications. In patients with repeated documented adherence issue, initiating device therapy may be more effective. When financial burden is the main barrier, initiating device-based therapies may be more cost effective in the long run. Unlike chronic recurrent costs incurred by drug therapies that may reach out-of-pocket cost of over $2,000 USD annually among Medicare patients,^4^ the cost of device-based therapies is a 1-time upfront expense. While the initial cost and implantation of devices is higher than drugs, the economic benefits of device therapy have the potential to surpass in the long run. For example, among eligible patients with HF, CRT has consistently been shown to be highly cost-effective when compared with medical therapy alone. The lower cost may, in part, be related to less need for urgent medical care (eg, emergency department visits or hospitalizations). It is important to note that these cost analyses were based on studies with a background of medical therapy.

Intensive treatment strategy with rapid up-titration of GDMT in patients with HF is safe and significantly improves outcomes.^5^ As devices target neurohormonal, autonomic, and structural abnormalities in HF, rapid device-based sequencing strategies may reverse these perturbances faster than the conventional approach and accelerate achieving clinical benefits. There are several shortcomings in the conventional approach of sequencing HF therapies and a more personalized approach should be advocated. While early use of devices in patients unable to achieve optimal GDMT is logical, there is a pressing need for trials dedicated to examining sequencing and rapid up-titration of all HF therapies, including drugs and devices, even in patients who are deemed tolerant of drugs. The residual risk in HF with reduced ejection fraction patients on optimal GDMT remains substantial necessitating novel therapeutic approaches. The implementation of such approaches necessitates significant efforts to address the high upfront cost of device therapy and ongoing cost of GDMT and facilitate an equitable accessibility and delivery of these therapies to patients (Figure 1).

**Figure 1:**
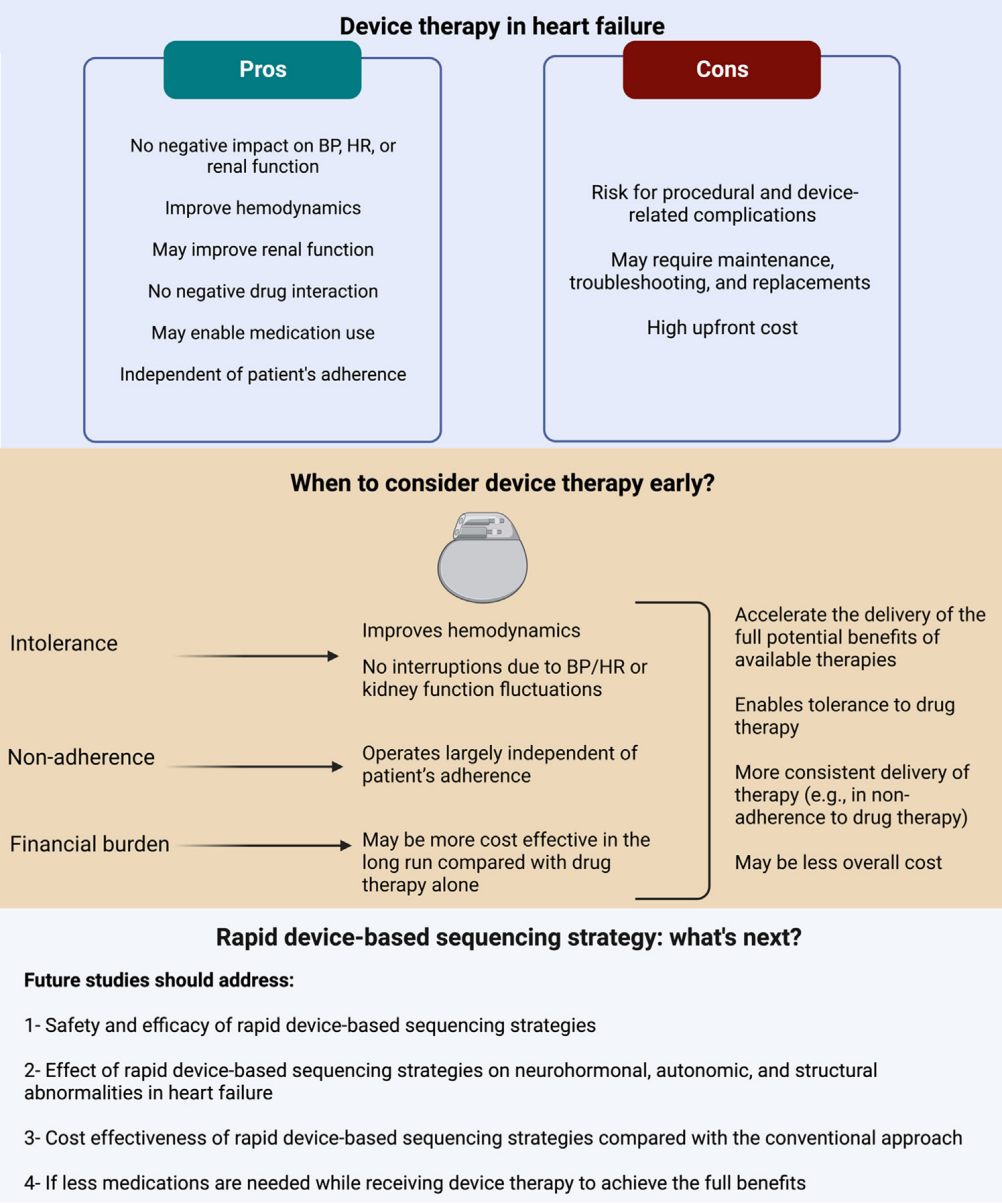
**Given the Shortcomings of the Conventional Approach to Heart Failure Therapies, a Rapid Medical and Device-Based Sequencing Strategies may be More Effective** Created with BioRender.com. BP = blood pressure; HR = heart rate.

## Funding support and author disclosures

Dr Butler is a consultant to Abbott, American Regent, Arca Biopharma, Amgen, Applied Therapeutic, AstraZeneca, Bayer, Boehringer Ingelheim, Bristol Myers Squibb, Cardiac Dimension, Cardior, CVRx, Cytokinetics, Edwards, Element Science, G3 Pharmaceutical, Innolife, Impulse Dynamics, Imbria, Inventiva, Lexicon, Lilly, LivaNova Janssen, Johnson and Johnson, Medtronics, Merck, Occlutech, Novartis, Novo Nordisk, Pfizer, Pharmacosmos, Pharmain, Roche, Sanofi, Sequana, SQ Innovation, 3live, and Vifor. Dr Fudim was supported by the 10.13039/100000050National Heart, Lung, and Blood Institute (NHLBI) (K23HL151744), the 10.13039/100000968American Heart Association (20IPA35310955), Mario Family Award, Duke Chair’s Award, Translating Duke Health Award, 10.13039/100004326Bayer, Bodyport and BTG Specialty Pharmaceuticals; and he has received consulting fees from Abbott, AxonTherapies, Bodyguide, Bodyport, Boston Scientific, CVRx, Daxor, Edwards LifeSciences, Feldschuh Foundation, Fire1, Gradient, Inovise Medical, Intershunt, NXT Biomedical, Pharmacosmos, PreHealth, Splendo, Vironix, Viscardia, Zoll. Dr Salah has reported that he has no relationships relevant to the contents of this paper to disclose.

## References

[1] Greene S.J., Fonarow G.C., DeVore A.D. (2019). Titration of medical therapy for heart failure with reduced ejection fraction. J Am Coll Cardiol.

[2] McCullough P.A., Mehta H.S., Barker C.M. (2021). Mortality and guideline-directed medical therapy in real-world heart failure patients with reduced ejection fraction. Clin Cardiol.

[3] Hariharaputhiran S., Peng Y., Ngo L. (2022). Long-term survival and life expectancy following an acute heart failure hospitalization in Australia and New Zealand. Eur J Heart Fail.

[4] Faridi K.F., Dayoub E.J., Ross J.S., Dhruva S.S., Ahmad T., Desai N.R. (2022). Medicare coverage and out-of-pocket costs of quadruple drug therapy for heart failure. J Am Coll Cardiol.

[5] Mebazaa A., Davison B., Chioncel O. (2022). Safety, tolerability and efficacy of up-titration of guideline-directed medical therapies for acute heart failure (STRONG-HF): a multinational, open-label, randomised, trial. Lancet.

